# Genome-Wide Identification of Pineapple AcINH Genes and Functional Characterization of *AcINH3* in Sucrose Metabolism and Drought Tolerance

**DOI:** 10.3390/plants15091306

**Published:** 2026-04-24

**Authors:** Yuyao Gao, Shanshan Huo, Anping Guo, Xiumei Zhang, Weisheng Sun, Wentian Xu, Hui Zhao, Qingsong Wu

**Affiliations:** 1 Key Laboratory of Tropical Fruit Biology, Ministry of Agriculture & Rural Affairs, Hainan Province Center of Pineapple Germplasm Innovation and Utilization Engineering Technology Research, Key Laboratory of Postharvest Physiology and Technology of Tropical Horticultural Products of Hainan Province, South Subtropical Crops Research Institute, Chinese Academy of Tropical Agricultural Sciences, Zhanjiang 524091, China; gaoyuyao@catas.cn (Y.G.); asiazhang1975@163.com (X.Z.); sunweisheng1234@sina.com (W.S.); xuwentian@catas.cn (W.X.); 2 Hainan Key Laboratory for Biosafety Monitoring and Molecular Breeding in Off-Season Reproduction Regions, Institute of Tropical Bioscience and Biotechnology & Sanya Research Institute, Chinese Academy of Tropical Agricultural Sciences, Sanya 572024, China; huoshanshan@itbb.org.cn (S.H.); gap211@126.com (A.G.)

**Keywords:** pineapple, AcINH3, CWIN activity, sucrose metabolism, drought stress

## Abstract

Seasonal drought constitutes a major abiotic stress limiting the growth and yield of pineapple, a globally important Crassulacean acid metabolism (CAM) crop. The sucrose catabolism mediated by cell wall invertase (CWIN) plays a vital role in regulating plant growth and development, as well as adaptive responses to abiotic stresses. Invertase inhibitors (INHs) serve as specific post-translational regulators that modulate CWIN enzymatic activity. However, the *INH* family has not been systematically characterized in pineapple, and its functional roles in mediating sucrose metabolism and drought resistance remain elusive. In this study, three *AcINHs* were identified from the pineapple genome, followed by comprehensive analyses of their gene structures, phylogenetic relationships, homology characteristics and protein structures. Structural analysis revealed that all *AcINH* members harbor conserved motifs 1, 2, 3, 5 and 9, whereas only *AcINH3* possesses motif 7. Expression analysis showed that only *AcINH3* was significantly transcriptionally induced by drought stress among all family members. Functional validation demonstrated that *AcINH3* knockout markedly elevated CWIN activity in pineapple seedling leaves, facilitating hexose accumulation and promoting plant growth and development. Moreover, *AcINH3*-edited lines exhibited enhanced drought resistance, accompanied by increased accumulation of soluble sugars (sucrose, glucose, fructose), abscisic acid (ABA), and proline (PRO), reduced malondialdehyde (MDA) content, and enhanced peroxidase (POD) activity. Biochemical assays further verified a direct physical interaction between AcINH3 and AcCWIN1, which mediates sucrose metabolism and drought stress responses. Collectively, this study identifies a novel AcINH3–AcCWIN1 post-translational module that modulates sugar metabolism and drought tolerance in pineapple, providing critical mechanistic insights for CAM plants. Our findings highlight *AcINH3* as a promising target for genome-editing breeding to enhance drought resistance in CAM crops.

## 1. Introduction

Pineapple (*Ananas comosus* L.) is one of the most economically important tropical crops worldwide, with an annual economic value of approximately $9 billion [1]. Beyond fresh consumption, pineapple is widely used in producing juice, vinegar, wine, citric acid, and bromelain [2]. Global demand for pineapple yield and quality has continuously increased [3]. China is a major global producer of pineapple. With ongoing global warming and climate change, the frequency and severity of seasonal droughts have increased in major pineapple cultivation areas, restricting plant growth and development while reducing fruit yield and quality [4]. Consequently, exploring the genetic control factors and regulatory mechanisms of pineapple in response to drought stress is crucial for breeding drought-resistant pineapple varieties, thereby supporting the development of high-quality and high-value pineapple production.

Plants respond to drought via diverse morphological, physiological, and biochemical adaptations, including improved root growth, stomatal closure, ABA production, and accumulation of proline and soluble sugars [5]. Soluble sugars (sucrose, glucose, fructose) act as vital osmoprotectants and signaling molecules, contributing to osmotic adjustment, membrane stabilization, ROS scavenging, and stress signal transduction [6,7,8]. Increased sugar accumulation enhances membrane stability and drought tolerance in plants [9,10]. Sucrose accumulation is closely associated with acid invertase activity, which has a direct impact on both flavor quality and stress resistance in fruits [11,12]. Based on their pH, the invertase enzymes are usually classified into neutral/alkaline invertases (A/N-INVs) and acid invertases (Ac-INVs), and Ac-INVs can be further divided into cell wall invertase (CWIN) and vacuolar invertase (VIN) [13,14]. CWIN catalyzes the irreversible hydrolysis of apoplastic sucrose and sustains photosynthate supply by regulating sink strength, which is critical for plant growth, development, and abiotic stress responses [15,16]. Previous studies demonstrated that CWIN inhibition represses growth in carrots [17], causes seed sterility in maize under drought [18], and leads to fruit/seed sterility in tomato [19]. Moreover, *CWIN* overexpression promotes hexose accumulation in citrus fruits [12] and enhances drought tolerance by elevating CWIN activity in tomato [20].

CWIN proteins exhibit inherent stability due to glycosylation modifications [21]. In addition to regulation at the transcriptional and post-transcriptional levels, accumulating evidence suggests that CWIN is predominantly inhibited post-translationally by its inhibitor proteins (INHs). Classified under the invertase/pectin methylesterase inhibitor (PMEI) superfamily, INHs constitute a family of non-glycosylated small proteins characterized by conserved pectin methylesterase inhibitor domains [22]. These inhibitors primarily engage in protein–protein interactions with invertase, thereby regulating its activity at the post-translational level [23]. Silencing *INVINH1* did not alter the transcription or protein levels of the cell wall invertase gene *LIN5*, yet it significantly increased CWIN activity in tomato fruits and seeds, resulting in higher seed weight and elevated hexose levels in the fruits [24]. This discovery provides the first evidence of the pivotal role of post-translational regulation of CWIN in the growth and development of tomato plants. This post-translational regulation of CWIN was subsequently confirmed to play a significant role in seed and tuber development in various plant species, including *Arabidopsis*, soybean, potato and litchi [25,26,27,28]. To date, evidence supporting the involvement of the INH–CWIN regulatory module in abiotic stress responses remains extremely limited. While INH function has been well defined in model plants, the INH family in pineapple has not been systematically characterized, and its roles in regulating seedling drought tolerance remain largely unknown.

Pineapple, a globally important CAM crop, suffers severe yield losses due to frequent seasonal drought stress exacerbated by climate change. Exploring key regulators involved in sugar metabolism and drought adaptation is critical for improving drought resistance and agricultural productivity. Currently, researchers have explored genetic improvement in pineapple [29], resulting in transgenic pineapple calluses with a red pigmentation in the adventitious shoots and calluses and transgenic plants with improved salt tolerance through gene overexpression [30,31]. As a versatile, efficient and powerful breeding tool, the CRISPR/Cas9-based genome editing technology has been widely used in the genetic improvement of many crops [32,33], while its application remains limited in pineapple research. While the INH–CWIN module is known to regulate plant growth and development, its biological function and molecular mechanism under drought stress remain largely unclear. To date, the characteristics and physiological roles of pineapple *AcINHs* in drought tolerance have not been systematically reported, and there are no reports on the phenotypic outcomes resulting from modifications in the expression of endogenous INHs in plants through gene editing technology.

In this study, three *AcINHs* were identified in pineapple, followed by comprehensive characterization of their gene structures, phylogenetic relationships, homology features and protein structures. We further profiled the expression patterns of *AcINH* family members across diverse tissues and under drought stress. Using CRISPR/Cas9-mediated gene editing, we demonstrated that *AcINH3* knockout markedly promoted sucrose catabolism and plant growth, and enhanced drought resistance in pineapple, which was primarily attributed to elevated CWIN activity. Additionally, the physical interaction between AcINH3 and AcCWIN1 was verified via yeast two-hybrid (Y2H) and glutathione S-transferase (GST) pull-down assays. Collectively, our findings uncover a novel regulator linking sucrose metabolism and drought stress responses in pineapple, and AcINH3 represents a promising molecular target for modulating sucrose metabolism and improving drought tolerance in CAM plants.

## 2. Results

### 2.1. Identification and Phylogenetic Analysis of AcINHs in Pineapple

**Figure 1:**
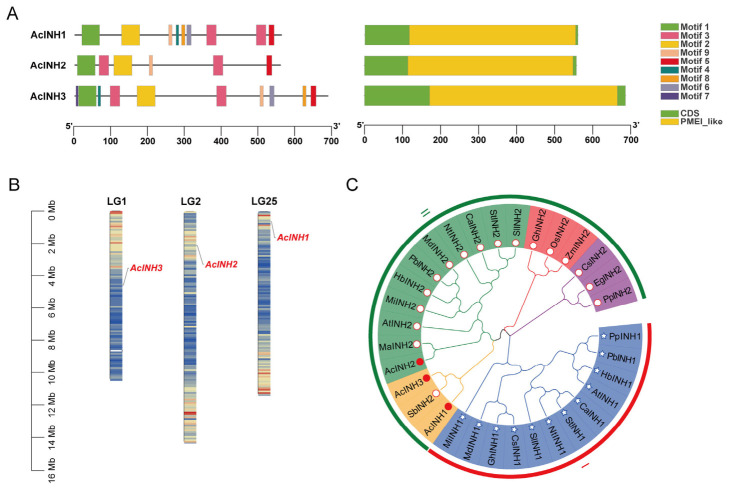
Characterization analysis of AcINHs in pineapple. (**A**) Conserved domains, gene structure, and motifs of AcINHs. (**B**) Chromosomal localization of the AcINH family genes in pineapple. (**C**) Phylogenetic tree of INH proteins in pineapple and other plants. The analysis comprised three AcINHs (*Ananas comosus*), two NtINHs (*Nicotiana tabacum*), two CaINHs (*Capsicum annuum*), two SlINHs (*Solanum lycopersicum*), two StINHs (*Solanum tuberosum*), two CsINHs (*Citrus sinensis*), two AtINHs (*Arabidopsis thaliana*), two MiINHs (*Mangifera indica*), two PbINHs (*Pyrus bretschneideri*), two HbINHs (*Hevea brasiliensis*), two PpINHs (*Prunus persica*), two GhINHs (*Gossypium hirsutum*), two MdINHs *(Malus domestica*), one OsINH (*Oryza sativa*), one ZmINH (*Zea mays*), one SbINH (*Sorghum bicolor*), one EgINH (*Elaeis guineensis*), and one MaINH (*Musa acuminata* subsp. *malaccensis*).

Genome-wide identification of AcINH family members in pineapple was carried out using the Pineapple Genomics Database (PGD) and BlastP for homologous searching, followed by comprehensive verification through homologous sequence alignment, sequence feature analysis, conserved domain identification, and three-dimensional protein structure modeling. Finally, three members of the INH family (*AcINH1*, *AcINH2*, and *AcINH3*) were identified. The detailed physicochemical characteristics of these genes and their encoded proteins, including gene ID, chromosomal location, isoelectric point, molecular weight, GRAVY value, and protein length, are summarized in Table S1. The AcINHs belong to the INVI/PMEI superfamily and possess a characteristic PMEI conserved domain, with their gene structure notably lacking introns (Figure 1A). Motif analysis revealed that the highly conserved motifs 1, 2, 3, 5, and 9 were present in all AcINHs. Motifs 4, 6, and 8 were specifically detected in both AcINH1 and AcINH3. Notably, motif 7 was only found in AcINH3, whereas nearly half of the motifs were absent in AcINH2 (Figure 1A). The three AcINHs were situated on distinct chromosomes: AcINH1 on chromosome 25, AcINH2 on chromosome 2, and AcINH3 on chromosome 1 (Figure 1B).

In this study, we used the INH amino acid sequences of pineapple and seventeen plant species, including rice, corn, sorghum, tobacco, Arabidopsis, tomato, apple, and pear, for phylogenetic tree analysis (Figure 1C). The INH proteins from these plants could be categorized into two groups, I and II, based on the different types of INH1 and INH2. AcINH1 and AcINH3 exhibited a close phylogenetic relationship with these two groups. Furthermore, AcINH3 clustered with AcINH2 and SbINH2 into one distinct subcluster, which formed a larger clade with INH2 from other species.

### 2.2. Homology Analysis of AcINHs

**Figure 2:**
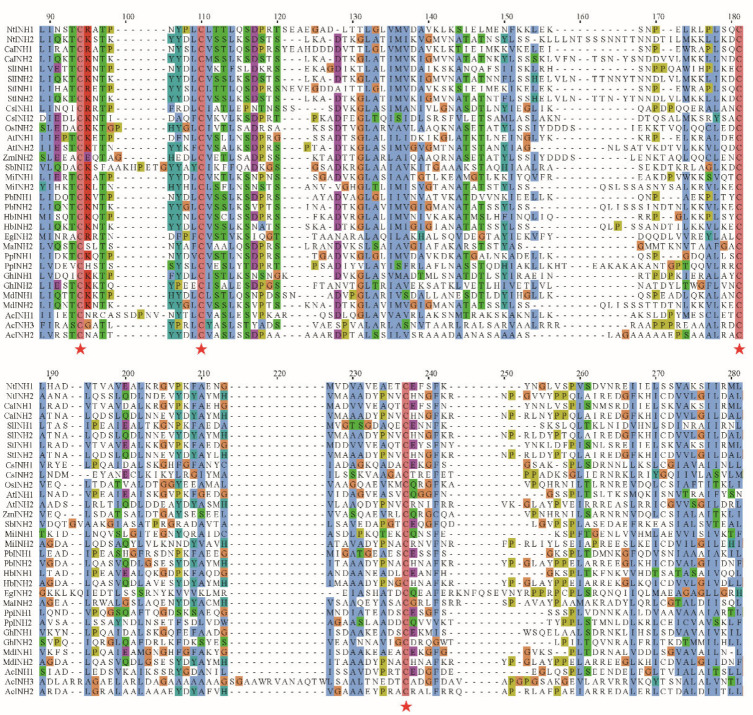
Homology comparison of AcINH amino acid sequences. The amino acid sequences of AcINHs from pineapple were analyzed in comparison to those from 17 other plant species. The four highly conserved cysteine residues characteristic of INH family proteins were highlighted using red five-pointed stars.

To investigate the sequence conservation, structural characteristics and evolutionary relationships of pineapple AcINH proteins and their homologous proteins from other plant species, we performed a comparative homology analysis of AcINH amino acid sequences from pineapple with those from the seventeen other plant species (Figure 2). The findings indicate that all proteins exhibited the four conserved cysteine residues located at positions 94, 110, 181, and 237, which are characteristic of the INH protein family. The two intermolecular disulfide bonds formed by these cysteine residues are crucial for maintaining the structural conformation of INH proteins [34]. The amino acid sequence homology of INH proteins across plants was low, and there was significant variability in INH amino acid sequences within individual plants. For example, the highest sequence homology observed among the three AcINHs in pineapple was only 32.6% (Table S2).

### 2.3. Protein Structure Prediction of AcINHs in Pineapple

The secondary structures of AcINH proteins were analyzed with the PRABI-GERLAND online server. Among the three AcINH proteins, the α-helix represented the most abundant secondary structural element, accounting for 50–70% of the total structure, followed by random coil at 20–35%. By contrast, the proportions of β-sheets and extended strands were relatively low, both accounting for less than 10% (Table S3).

**Figure 3:**
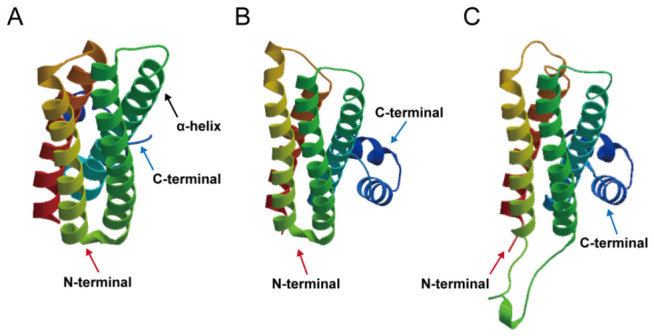
Tertiary structure of AcINH proteins. (**A**) Tertiary structure of AcINH1 protein. (**B**) Tertiary structure of AcINH2 protein. (**C**) Tertiary structure of AcINH3 protein.

The tertiary structure models of AcINH proteins were constructed using the online server SWISS-MODEL. Structural analysis revealed that the N-terminal region of AcINH proteins contained four asymmetric α-helices that folded inward rather than extending outward (Figure 3). Each of these four α-helices harbors an AIN-binding motif, which acts as a key functional region involved in regulating AIN activity.

### 2.4. Expression Patterns of AcINHs in Pineapple

**Figure 4:**
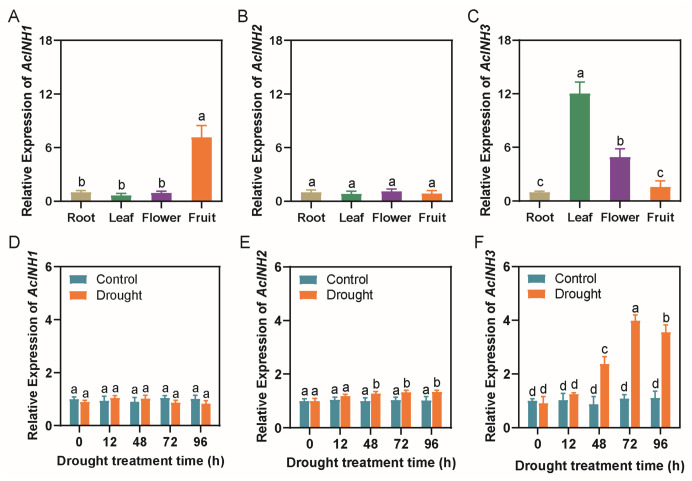
Analysis of *AcINH* expression in different tissues and under drought treatment. (**A**) Analysis of *AcINH1* expression in different tissues. (**B**) Analysis of *AcINH2* expression in different tissues. (**C**) Analysis of *AcINH3* expression in different tissues. (**D**) Analysis of *AcINH1* expression under drought treatment. (**E**) Analysis of *AcINH2* expression under drought treatment. (**F**) Analysis of *AcINH3* expression under drought treatment. Bars represent mean ± SD (*n* = 3), and a–d represent *p* ≤ 0.05 (one-way ANOVA with Tukey’s multiple comparison test).

To investigate the potential functional roles of *AcINHs*, we examined the expression patterns of the three *AcINHs* across various pineapple tissues (Figure 4A–C). *AcINH1* exhibited a pronounced fruit-specific expression pattern, with its transcript abundance being significantly higher in fruits than in roots, leaves, and flowers. *AcINH2* displayed constitutively low expression across all examined tissues. *AcINH3* showed a leaf- and flower-preferential expression profile, with the highest transcript abundance found in leaves, followed by flowers (Figure 4A–C). Collectively, the distinct tissue-specific expression patterns of the *AcINH* gene family suggest functional diversification, implying that these genes may execute specialized roles in the growth and development of different pineapple tissues.

To determine whether *AcINHs* respond to drought stress, we analyzed the expression levels of the three genes in the leaves of pineapple seedlings under PEG-simulated drought stress at various treatment durations. The results indicated that the expression of *AcINH1* and *AcINH2* remained relatively stable and did not exhibit significant changes with prolonged treatment time (Figure 4D,E). In contrast, the expression of *AcINH3* initially increased before subsequently declining, reaching a peak at 72 h, and was significantly higher than that of the control group at 48 h, 72 h, and 96 h (Figure 4F). This suggests that *AcINH3* plays a critical role in the response of pineapple to drought stress. Collectively, these results reveal functional divergence among the *AcINH* gene family in response to drought stress, with *AcINH3* being the only member significantly induced by drought. Thus, we then selected *AcINH3* for biochemical role and functional analyses in pineapple plants.

### 2.5. Knockout of AcINH3 in Pineapple Enhances CWIN Activity, Sucrose Catabolism, and Plant Growth

**Figure 5:**
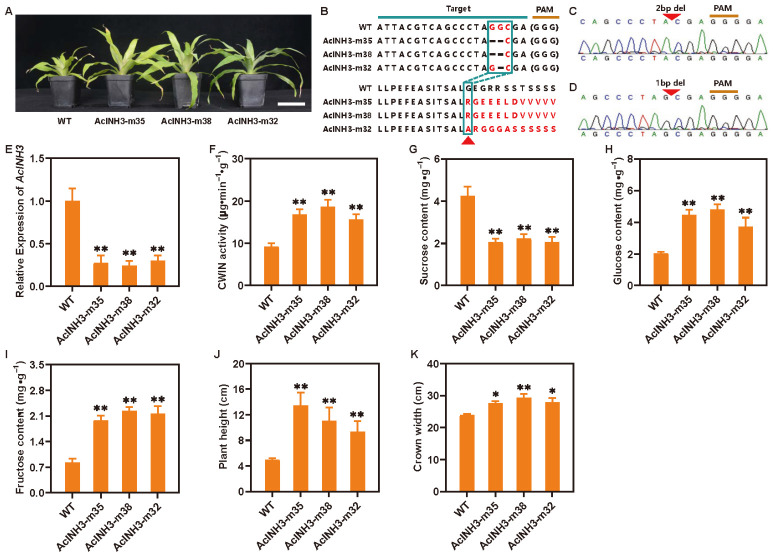
*AcINH3* regulates sucrose metabolism and enhances growth by modulating CWIN activity. (**A**) Morphological characteristics of GM plants, including *AcINH3-m35*, *AcINH3-m38*, and *AcINH3-m32*. The scale bar indicates 5 cm. (**B**) Target sequence analysis of GM plants. (**C**,**D**) The edited types of GM plants. (**E**) *AcINH3* expression in GM plants. (**F**) CWIN activity of GM plants. (**G**) Sucrose content of GM plants. (**H**) Glucose content of GM plants. (**I**) Fructose content of GM plants. (**J**) Plant height of GM plants. (**K**) Crown width of GM plants. Bars represent mean ± SD (*n* = 3), and * and ** represent *p* ≤ 0.05 and *p* ≤ 0.01 (one-way ANOVA with Tukey’s multiple comparison test). 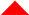 denotes the 29th amino acid. 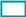 indicates the position at which the nucleotide base and the corresponding amino acid begin to undergo mutation.

A CRISPR/Cas9 editing vector targeting *AcINH3* was constructed using the Golden Gate cloning system. The process commenced with the cloning of the *AcINH3* cDNA, followed by the design of the sgRNA for *AcINH3* based on the conserved sequences of *AcINHs*. The sgRNA sequence (attacgtcagccctaggcga) (Figure 5B) was selected due to its optimal specificity, high editing efficiency, and lack of off-target effects. Subsequently, editing vectors suitable for monocotyledonous pineapple were screened, and target primers were designed using the CRISPR-multiplex tool. The construction of the *AcINH3* editing vector was successfully achieved through enzyme digestion and ligation techniques. Furthermore, *Agrobacterium*-mediated transformation was performed in pineapple embryogenic calli, resulting in the generation of *AcINH3*-edited or genetically modified (GM) plants with three strains: *AcINH3-m35*, *AcINH3-m38*, and *AcINH3-m32* (Figure 5A).

To determine the successful editing of *AcINH3*, the type of editing, and the genotype of the GM plants, sequencing was employed. Sequencing results indicated that the strains *AcINH3-m35* and *AcINH3-m38* demonstrated homozygous mutations characterized by the deletion of two bases, leading to frameshift mutations in the encoded amino acids beginning at the 29th position (Figure 5B,C). In addition, the strain *AcINH3-m32* exhibited homozygous mutations characterized by the deletion of a single base, resulting in six consecutive amino acid alterations starting from the 29th position (Figure 5B,D).

Further analysis revealed a significant decrease in *AcINH3* expression in GM plants (Figure 5E). Notably, CWIN activity and hexose levels (glucose and fructose) in GM plants were significantly elevated compared to WT plants (Figure 5F,H,I), while sucrose content was significantly reduced (Figure 5G). These findings suggested that *AcINH3* knockout markedly enhanced CWIN activity and facilitated the conversion of sucrose to hexose. From a plant growth perspective, GM plants demonstrated significantly more robust growth and development compared to WT plants (Figure 5A), with both the width and height of GM plants being significantly greater than those of WT plants (Figure 5J,K). In summary, *AcINH3* knockout substantially promoted the growth and development of pineapple plants, likely due to the increased CWIN activity in GM plants, which enhances the conversion of sucrose to hexose, thereby providing an ample carbon source and energy for the growth and development of pineapple plants. This in vivo study provides empirical evidence supporting the hypothesis that *AcINH3* regulates sucrose metabolism by modulating CWIN activity.

### 2.6. Knockout of AcINH3 in Pineapple Promotes Drought Resistance

**Figure 6:**
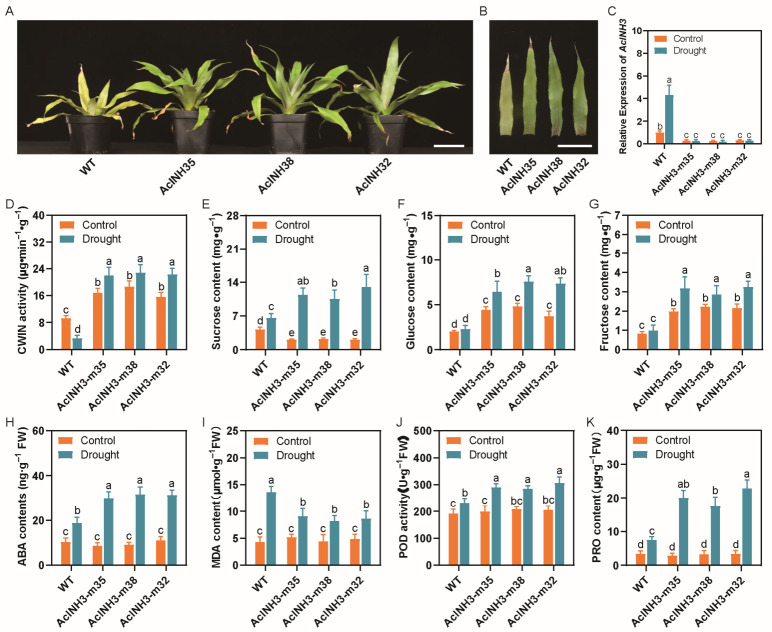
Determination of drought resistance in GM plants. (**A**) Morphological characteristics of GM plants. The scale bar indicates 5 cm. (**B**) Leaf morphology of GM plants. The scale bar indicates 5 cm. (**C**) *AcINH3* expression in WT and GM plants under drought stress. (**D**) CWIN activity in WT and GM plants under drought stress. (**E**) Sucrose content in WT and GM plants under drought stress. (**F**) Glucose content in WT and GM plants under drought stress. (**G**) Fructose content in WT and GM plants under drought stress. (**H**) ABA content in WT and GM plants under drought stress. (**I**) MDA levels in WT and GM plants under drought stress. (**J**) POD activity in WT and GM plants under drought stress. (**K**) PRO levels in WT and GM plants under drought stress. Bars represent mean ± SD (*n* = 3), and a–e represent *p* ≤ 0.05 (one-way ANOVA with Tukey’s multiple comparison test).

Following a 72 h treatment with 20% PEG6000, it was unexpectedly observed that the WT plants exhibited pronounced chlorosis, and the tips of the outer leaves dried inward. In contrast, GM plants demonstrated vigorous growth, with no signs of chlorosis or leaf tip desiccation (Figure 6A,B), thereby demonstrating enhanced drought resistance compared to WT plants.

Under drought stress conditions, the expression level of *AcINH3* in WT plants increased significantly, whereas it remained extremely low in GM plants, showing no significant deviation from the control (Figure 6C). Notably, CWIN activity exhibited an inverse relationship with the expression level of *AcINH3*; it was significantly diminished in WT plants while markedly enhanced in GM plants (Figure 6D). These results suggest that *AcINH3* is upregulated in response to drought stress; the expression of *AcINH3* increases in WT plants, leading to a decrease in CWIN activity due to the inhibitory effects of AcINH3. Conversely, the expression level of *AcINH3* remains extremely low in GM plants, resulting in negligible inhibitory effects, which allows for an increase in CWIN activity.

In our study, we observed that under drought stress conditions, the sucrose content in WT plants exhibited a significant increase, whereas the levels of glucose and fructose did not differ significantly from those in the control group. In contrast, GM plants demonstrated a marked increase in the content of sucrose, glucose, and fructose (Figure 6E–G).

We further assessed the indicators of drought resistance. The findings indicated that, under drought stress conditions, the levels of ABA, MDA, PRO, and the activity of POD in both WT and GM plants increased rapidly, exhibiting significantly higher values compared to the control group. Notably, the ABA and PRO levels, as well as POD activity, were markedly elevated in GM plants relative to WT plants (Figure 6H,J,K), whereas the MDA content was significantly reduced in GM plants compared to WT plants (Figure 6I). These findings suggest that *AcINH3* knockout substantially enhances drought resistance in pineapple plants.

### 2.7. AcINH3 Interacts with AcCWIN1 In Vitro

**Figure 7:**
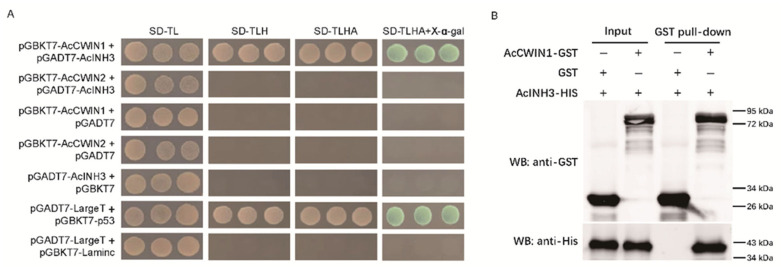
Interaction analysis of AcINH3 and AcCWINs in vivo. (**A**) Interaction analysis of AcINH3 and AcCWINs by the yeast two-hybrid system. Yeast cells were co-transformed with pGBKT7-AcCWIN1/2 and pGADT7-AcINH3; the pGADT7-LargeT and pGBKT7-p53 served as positive controls, and pGADT7-LargeT and pGBKT7-Laminc were used as negative controls. Cultures were grown in SD-TL, SD-TLH, and SD-TLHA media, respectively. The interaction was detected only on pGBKT7-AcCWIN1 and pGADT7-AcINH3 co-cultured medium. (**B**) Interaction analysis of AcINH3 and AcCWINs by glutathione S-transferase pull-down. AcCWIN1-GST and AcINH3-HIS plasmids were transformed into BL21 receptor cells. Following induction, extraction, and purification, both AcCWIN1-GST and AcINH3-HIS proteins were detected in the input assay. However, the AcINH3-HIS protein was identified in the precipitate of AcCWIN1-GST following a GST pull-down assay.

Previous research indicated that INHs and CWINs have physical interactions in vivo, which affect the activity of CWIN [24]. To identify the interacting partners of pineapple AcINH3, a Y2H assay was performed to investigate the physical interaction between AcINH3 and two known AcCWIN isoforms (AcCWIN1 and AcCWIN2) [11] (Table S4). AcCWIN1 and AcCWIN2 were used as “bait,” while AcINH3 functioned as “prey” for point-to-point verification of protein interactions. The results indicated that AcINH3 specifically interacted with AcCWIN1, whereas no interaction was observed between AcINH3 and AcCWIN2. Additionally, the control group yeast strains, pGBKT7-AcCWIN1, pGBKT7-AcCWIN2, and pGADT7-AcINH3, did not display spontaneous activation or cytotoxic effects (Figure 7A).

The interaction between AcCWIN1 and AcINH3 was further confirmed by a GST pull-down assay. Both AcCWIN1-GST and AcINH3-HIS proteins were expressed, as evidenced by their detection in the input samples. Furthermore, AcINH3-HIS was observed in the precipitate of AcCWIN1-GST, while it was absent in the control GST protein without the target (Figure 7B). Collectively, these findings from Y2H and GST pull-down assays provide compelling evidence for the direct interaction between AcINH3 and AcCWIN1.

## 3. Discussion

### 3.1. AcINH3 Influences Sucrose Metabolism by Regulating CWIN Activity

Beyond transcriptional regulation, CWIN activity in horticultural crops is modulated by INHs at the post-translational level through protein–protein interactions. In tomato, INVINH1 interacts with CWIN in the cell wall matrix, and silencing *INVINH1* significantly increases CWIN activity and elevates hexose accumulation in fruits [24]. In potato, StInvInh1 interacts with StCWINV2, specifically modulating CWIN activity without altering its expression level [27]. In this study, *AcINH3*, which exhibits a strong response to drought stress, was identified in pineapple. The interaction between AcINH3 and AcCWIN1 was confirmed by Y2H and pull-down assays. Following *AcINH3* knockout using CRISPR/Cas9 gene editing technology, a significant enhancement in CWIN activity was observed, accompanied by a decrease in sucrose content and an increase in hexose content in GM lines. These findings are consistent with those of previous studies [24,26]. Thus, similar to other horticultural crops, AcINH3 in pineapple regulates CWIN activity through protein–protein interactions, thereby influencing sugar accumulation.

### 3.2. AcINH3 Negatively Regulates the Growth and Development of Pineapple

The INH–CWIN regulatory module influences the rate of sucrose decomposition, subsequently altering sugar composition and content, which is essential for plant growth and development [35]. For instance, the overexpression of *INHs* in *Arabidopsis* and tobacco suppresses invertase activity, impedes plant growth and development, and ultimately results in plant mortality [36]. In tomato, silencing *INVINH1* did not impact the transcription of cell wall invertase genes *LIN6* and *LIN8*, but it significantly enhanced CWIN activity and delayed leaf senescence [24]. These findings underscore the crucial role of INHs in plant growth and development. In this study, CRISPR/Cas9 gene editing technology was used to knock out *AcINH3*, resulting in a marked enhancement of CWIN activity in GM plants, which exhibited robust growth. The results support the hypothesis that AcINH3 plays a crucial role in sucrose metabolism and pineapple growth and development, and that eliminating AcINH3 effectively promotes pineapple growth.

### 3.3. AcINH3 Negatively Modulates Drought Tolerance in Pineapple

Under adverse environmental conditions, plants primarily modulate intracellular sugar accumulation via key regulatory factors involved in glucose metabolism to mitigate stress responses [37]. For instance, changes in the expression of INHs significantly influenced abiotic stress resistance in species such as potato [38], tomato [39], poplar [40], and *Arabidopsis* [41]. Notably, distinct INHs perform diverse functions in managing abiotic stress. The suppression of *INVINH1* improved CWIN activity and cold tolerance in tomato [39], whereas *Zm-INVINH4* is significantly expressed under drought stress, accompanied by a decrease in CWIN activity in maize [42]. Nonetheless, the role of INHs in drought stress resistance in plants remains inadequately understood. Under conditions of drought stress, plant cells typically synthesize and accumulate osmotic substances rapidly, thereby regulating osmotic pressure to mitigate cell damage caused by water scarcity [8]. Drought stress can enhance the accumulation of soluble sugars by increasing sink strength, with CWIN playing a crucial role in modulating this sink strength through the regulation of sucrose allocation to the extracellular matrix [43]. Researchers have demonstrated that the hydrolysis of apoplastic sucrose, facilitated by CWIN, contributes to the generation of negative water potential and establishes a concentration gradient that supports the continuous export of sucrose to the apoplastic space [15,44]. The significantly enhanced CWIN activity in tomato plants led to enhanced drought stress tolerance [20]. In this study, *AcINH3* knockout activated CWIN activity, thereby enhancing soluble sugar accumulation and drought tolerance in pineapple. These results indicate that increased CWIN activity is the primary cause of improved drought resistance in *AcINH3* knockout lines. Furthermore, we speculate that intracellular sucrose resynthesis or the regulation of sugar transporters is also implicated in this soluble sugar accumulation and drought stress response [45], representing key directions for our future investigations. It is worth noting that *AcINH3* transcription was significantly reduced in gene-edited lines despite intact promoter regions, likely due to post-transcriptional regulation such as nonsense-mediated mRNA decay (NMD) and RNA instability induced by coding region mutations [46,47].

## 4. Materials and Methods

### 4.1. Bioinformatics Analysis

The gene structure of the AcINH family was analyzed using the Gene Structure Display Server v.2.0 online, while the amino acid sequences and homology of the AcINH family were assessed using MegAlign v.7.1 (DNASTAR Inc., Madison, WI, USA). MEME Suite v.5.5.3 was used to investigate the conserved domains and motifs present in AcINH family proteins. In addition, the genomic location information of AcINH family genes was extracted from the genome data of pineapple F153, which has a closer genetic relationship with ‘Comte de Paris’. The chromosomal distribution of these genes was visualized using the “Show Genes on Chromosome” function in TBtools (https://github.com/CJ-Chen/TBtools-II, 20 September 2024). In addition, genomic location information of the AcINH family genes was extracted from the genome data of the pineapple F153, which has a closer genetic relationship with ‘Comte de Paris’.

In the phylogenetic analysis, the amino acid sequences of INHs from pineapple, rice, corn, sorghum, tobacco, Arabidopsis, tomato, apple, and pear were retrieved from the Phytozome database (Table S5). These sequences were analyzed using MEGA v.7.0, applying the Jones–Taylor–Thornton model. Subsequently, a phylogenetic tree was constructed using the maximum likelihood method [48].

In addition, the secondary structures of AcINH proteins were analyzed via the PRABI-GERLAND online platform, and their tertiary structure models were constructed with the SWISS-MODEL online server.

### 4.2. Plant Materials

In this study, the conventional dominant variety ‘Comte de Paris’ pineapple in the “Queen” category was selected as the experimental material and cultivated in the pineapple germplasm resource nursery at the South Subtropical Crops Research Institute, Zhanjiang, China (21°100′ N, 110°160′ E). A standardized approach was used for fertilization, irrigation, and pest management using pot cultivation.

### 4.3. Drought Stress Treatment and Sample Collection

To investigate the expression patterns of *AcINHs*, we collected various tissues (roots, leaves, flowers, and fruits) from pineapple plants. Based on the water stress treatment method described by [49], pineapple seedlings at the ten-leaf stage underwent treatment with 20% PEG6000, while the control group was maintained in water. PEG treatment was performed once at the beginning of the drought stress experiment, and the state was maintained until sampling. Leaf samples were collected at 12, 24, 48, 72, and 96 h post-treatment, with each time point replicated three times. GM plants were also treated with 20% PEG6000 solution for 72 h. The collected leaves were frozen and stored at −80 °C for subsequent analyses of drought resistance indices, enzyme activity, sugar content, and gene expression.

### 4.4. qRT-PCR Analysis

Total RNA from pineapple leaves was extracted using the Polysaccharide Polyphenol Plant Total RNA Extraction Kit (Beijing Huayueyang Biotechnology Co., Ltd., Beijing, China). Subsequently, the TransScript One-Step gDNA Removal and cDNA Synthesis SuperMix kits (Beijing Quanshi Gold Biotechnology Co., Ltd., Beijing, China) were used for reverse transcription, facilitating first-strand cDNA synthesis. Primers were designed using Primer Premier v.5.0, with actin as the internal reference gene. qRT-PCR analysis was performed using the 2×TransStart^®^ Top Green qPCR SuperMix kit (TransGen Biotech, Beijing, China). Relative gene expression levels were determined using the 2^−△△Ct^ method. Primer information for gene expression analysis is provided in Table S6.

### 4.5. Construction of AcINH3 Editing Vector

The *AcINH3* editing vector was constructed using the Golden Gate cloning method. Initially, cDNA of *AcINH3* was cloned, and then the sgRNA of *AcINH3* was designed utilizing the online tool CRISPOR (http://crispor.tefor.net/, 20 September 2024). The sgRNA sequence (attacgtcagccctaggcga) located within the 70–89 base pair region of the *AcINH3* coding sequence was identified as the specific target for this study. Comparative analyses revealed that this target exhibits excellent specificity, high editing efficiency, and remarkably low off-target effects.

Subsequently, suitable vectors for pineapple were identified from the available options, specifically pMOD_A1110, pMOD_B2312, pMOD_C0000, and pTRANS_260d, using the CRISPR-multiplex online tool (https://github.com/eric-erki/awesome-CRISPR#multiplexing, 20 September 2024). Target primers were designed using the “Primer Design” and “Mapping” features of the CRISPR-multiplex tool (Table S7). The recombinant plasmid for *AcINH3* expression was constructed using enzyme digestion and ligation techniques [50].

### 4.6. Agrobacterium-Mediated Genetic Transformation

The CRISPR/Cas9 editing vector targeting *AcINH3* was constructed, and the pineapple embryogenic callus was transformed using *Agrobacterium*-mediated transformation [51]. Briefly, the initial callus was derived from the axillary buds of ‘Comte de Paris’ on a callus induction medium for approximately 30 to 40 days. Subsequently, these primary calli underwent proliferation culture and somatic embryo induction culture to develop into embryogenic callus, which served as transgenic receptor material. The embryogenic callus was sectioned into pieces measuring 0.8 to 1.0 cm and incubated in darkness on a pre-culture medium for two days prior to infection with Agrobacterium strains harboring the AcINH3 editing vector. Following infection with the bacterial suspension at an OD600 of 0.3 to 0.5 for 3 to 5 min, the pre-cultured embryogenic callus was maintained in darkness on a co-culture medium for three days. Selection of resistant buds and callus was conducted on a medium containing phosphinothricin, and the resistant buds were subsequently rooted on a rooting medium. The bar gene was screened using a phosphinothricin selection concentration of 1.5 mg·L^−1^. Various media used in the experiments are detailed in Table S8.

### 4.7. Identification and Genotype Analysis of GM Plants

Sequencing was employed to ascertain the successful editing of *AcINH3*, to identify the type of editing, and to determine the genotype of the GM plants. The synthesis of target primers (Table S9) and the detection of edited sequences were conducted by Shenggong Bioengineering Co., Ltd., Shanghai, China. The type of editing and the genotype of the GM plants were confirmed based on the sequencing results.

### 4.8. Evaluation of Off-Target Effects

To minimize off-target effects, we concentrated on analyzing the conserved sequences of *AcINHs* in the design of sgRNA for *AcINH3*, and we selected sequences that demonstrated high specificity, elevated editing efficiency, and no off-target effects as the target sgRNA. Additionally, the editing efficiency and potential off-target effects of the *AcINH3* can be rapidly assessed through sequencing.

### 4.9. CWIN Activity Assay

CWIN activity was assessed following a previously established protocol [52]. Each assay included three biological replicates and three technical replicates.

### 4.10. Determination of Sugar Content

The contents of sugars (fructose, glucose, and sucrose) were measured using high-performance liquid chromatography. To determine sugar content, chromatography was conducted using a Series 200 amino acid column at a column temperature of 35 °C, with a mobile phase consisting of acetonitrile and deionized water in a ratio of 70:30 (*v*/*v*). The flow rate was maintained at 1 mL·min^−1^, and a sample volume of 10 μL was used for each analysis. Quantitative determinations were conducted using the external standard method [53]. Qualitative and quantitative analyses were performed in accordance with the methodologies established by Wang et al. (2018) [54].

### 4.11. Determination of ABA Content and Antioxidant Enzyme Activity

The quantification of ABA content was performed following the methodology described by Šimura et al. (2018) [55]. Ground samples, weighing 50 milligrams each, were extracted using a 75% methanol solution, subsequently concentrated, and re-dissolved in an 80% methanol solution. The analysis was executed utilizing a liquid chromatography-electrospray ionization–tandem mass spectrometry (LC-ESI-MS/MS) system, specifically the UHPLC, ExionLC™ AD (SCIEX, Framingham, MA, USA). The mobile phase comprised solvent A, consisting of water with 0.04% acetic acid, and solvent B, consisting of acetonitrile with 0.04% acetic acid. The concentration of plant hormones was determined using a mass spectrometer (AB 6500+QTRAP^®^ LC-MS/MS System, SCIEX, Framingham, MA, USA) equipped with an ESI Turbo ion spray interface. The experiment was conducted with three biological replicates.

POD activity was assessed using the guaiacol method [56], MDA level was determined by the thiobarbituric acid colorimetric method [57], and PRO content was quantified through the ninhydrin colorimetric method [58].

### 4.12. Protein Interaction Analysis

In the Y2H assay, the cDNA sequences of AcCWIN1 and AcINH3 were amplified and inserted into the pGBKT7 or pGADT7 vectors. Initially, the plasmids pGBKT7-AcCWIN1, pGBKT7-AcINH3, and pGBKT7 (negative control) were introduced into yeast strain AH109 to assess self-activation. Subsequently, various combinations of plasmids, including pGBKT7-AcCWIN1 and pGBKT7-AcINH3, pGBKT7 and pGBKT7-AcINH3 (controls), pGBKT7-AcCWIN1 and pGBKT7 (trial controls), pGADT7-LargeT and pGBKT7-p53 (positive control), and pGADT7-LargeT and pGBKT7-LaminC (negative control), were co-cultured in SD-TL (-Trp/-Leu), SD-TLH (-Trp/-Leu/-His), and SD-TLHA (-Trp/-Leu/-His/-Ade) media for detection.

In the GST pull-down experiment, fusion expression vectors for AcCWIN1-GST and AcINH3-HIS were constructed. These vectors were extracted and transformed into *Escherichia coli* BL21(DE3) competent cells, followed by the selection of monoclonal colonies for induction culture. The AcCWIN1-GST and AcINH3-HIS proteins were then extracted and purified. Finally, pull-down assays and Western blot analyses were performed to elucidate the interaction mechanisms of the target proteins.

### 4.13. Statistical Analysis

Data analysis was performed with SAS v.9.3 (SAS Institute, Cary, NC, USA) using analysis of variance (PROC ANOVA) with multiple comparison correction. Mean separation was assessed using Duncan’s multiple range test at the 0.05 significance level. In addition, Spearman’s correlation analysis was performed to evaluate the associations among attributes, and the results were visualized in a heatmap.

## 5. Conclusions

This study represents the first systematic characterization of the AcINH gene family in pineapple, accompanied by profiling of its expression patterns under drought stress. Notably, AcINH functions as a crucial negative regulator modulating sucrose catabolism, plant growth and development, and drought responses in pineapple. Knockout of *AcINH3* elevated CWIN activity, reduced sucrose levels, promoted hexose accumulation, and subsequently boosted plant growth and development. Under drought stress, knockout of *AcINH3* enhanced soluble sugar (sucrose, glucose, fructose) accumulation, elevated ABA and PRO contents, and strengthened POD activity, thus conferring enhanced drought resistance. These findings establish *AcINH3* as a promising molecular target for modulating sugar metabolism and drought resistance in pineapple, providing key theoretical insights into CWIN-mediated stress tolerance and practical value for improving yield stability and fruit quality under global drought stress.

## Data Availability

All data included in the study were publicly available. All experiment data are provided in the attachment.

## Supplementary Materials

The following supporting information can be downloaded at: https://www.mdpi.com/article/10.3390/plants15091306/s1, Table S1: Information on *AcINHs* and physicochemical characteristics of AcINH proteins; Table S2: Homology analysis of AcINH protein sequences in pineapple; Table S3: Prediction of the secondary structures of AcINH proteins; Table S4: Information of the *CWIN* family in pineapple; Table S5: Amino acid sequences of INHs in eighteen plant species; Table S6: Primers for qRT-PCR detection of *AcINHs*; Table S7: Target primers for *AcINH3*; Table S8: Medium and formulation; Table S9: Primers for detection of *AcINH3* target.
